# Neuromechanical model of reflexes and locomotor rhythms in the crayfish leg

**DOI:** 10.1186/1471-2202-14-S1-P55

**Published:** 2013-07-08

**Authors:** Donald H Edwards, Bryce Chung, Julien Bacque-Cazenave, Daniel Cattaert

**Affiliations:** 1 Neuroscience Institute, Georgia State University, Atlanta, GA, 30302-5030 USA; 2 Inst. de Neurosciences Cognitives et Intégratives d'Aquitaine, Univ. of Bordeaux, 33405,Talence Cedex, France

## 

**Figure 1:**
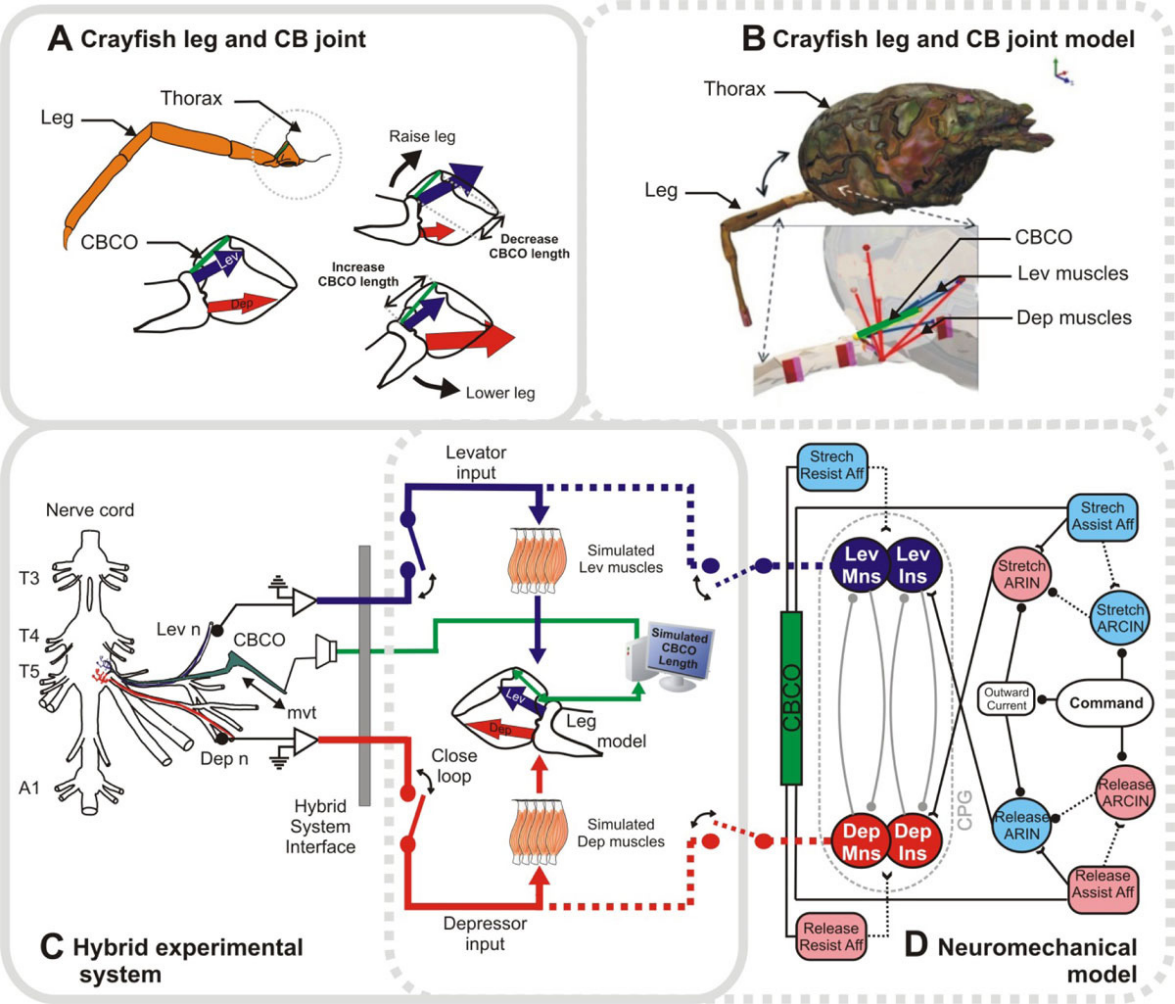
**Neuromechanical model**. Body model is at left, showing thorax and left 5^th ^leg with hinges, CBCO stretch receptor, and Dep (red) and Lev (blue) muscles identified. Circuit is at right, with Dep MNs, INs, and muscles in red, and Lev in blue. Muscles and CBCO correspond to those shown in body diagram.

The role of sensory feedback in the production of locomotor rhythms has been discussed for a hundred years [1], but a detailed description has emerged only recently with the coupling of neurophysiological and behavioral experiments and neuromechanical models [2]. In both cats and stick insects, this combination has helped clarify the role of proprioceptive feedback in determining the stance-swing transition during walking. The role of sensory feedback in posture and locomotor control of the crayfish leg has been intensively investigated over the last 25 years, and the neural circuitry governing leg elevation and depression movements during static posture and walking has been described [3]. We have developed a hybrid neuromechanical experiment consisting of an AnimatLab neuromechanical model (http://www.animatlab.com) of the crayfish thorax and 5^th ^leg linked to the corresponding sensory and motor nerves of the living crayfish thoracic nervous system. We found that lifting the model leg would evoke resistance reflexes in tonically active preparations similar to those seen in intact resting animals, whereas the reflex reversed sign to become assistive during a locomotor rhythm. Moreover, the rhythm had a much higher frequency with the sensory feedback closed than when the loop was opened. To help determine whether the known circuitry can account for postural reflexes and reflex-assisted walking, we linked the same neuromechanical model of the crayfish leg to a model of the neural circuit that controls leg levation and depression (Figure 1). We found that an appropriately 'tuned' model could replicate the hybrid experimental results, and that this depended on the proper balance between the strength (gain) of the assistance reflex and the strength and stability of the half-center oscillator. To increase the intrinsic CPG frequency, the leg levation assistance reflex had to be sufficiently sensitive and strong to reset the CPG earlier in the interval between depression and levation motor bursts. The CPG had to be sufficiently robust to produce a stable low-frequency burst pattern without reflex input, but be able to reset in response to appropriately timed input.

**Figure 2:**
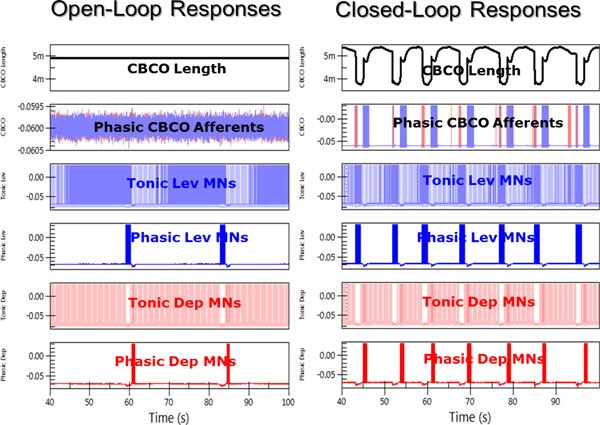
**Model responses under open loop (left) and closed loop (right) conditions**. Closing the feedback loop increased the burst frequency of the Lev/Dep CPG.
